# Development and Validation of a Neuro-Intensive Care Protocol for Traumatic Brain Injury Management

**DOI:** 10.7759/cureus.79566

**Published:** 2025-02-24

**Authors:** Sindu K Mathew, Aruna S, Ramesh C Vasudevan, Vivek V, Porkodi Arjunan

**Affiliations:** 1 Medical-Surgical Nursing, College of Nursing, Thalassery, Thalassery, IND; 2 Nursing, Sri Ramachandra Institute of Higher Education and Research, Chennai, IND; 3 Neurosurgery, Tellicherry Cooperative Hospital, Thalassery, IND; 4 Neurosurgery, Sri Ramachandra Institute of Higher Education and Research, Chennai, IND; 5 Medical-Surgical Nursing, College of Applied Medical Sciences, King Faisal University, Hofuf, SAU

**Keywords:** consensus protocol traumatic brain injury, critical care management, delphi method, development and validation of tbi management protocol, evidence-based management, neurocritical care, neurocritical care clinical practice guideline traumatic brain injury, neuro-intensive care protocol (nicp), traumatic brain injury management protocol, traumatic brain injury (tbi)

## Abstract

Introduction

Traumatic brain injury (TBI) is a global public health concern with high mortality and disability rates, particularly among younger populations. Structured treatment protocols might improve outcomes and reduce mortality. This study aims to develop and validate the Neuro-Intensive Care Protocol for TBI (NICP-TBI) using the Medical Research Council framework for designing and evaluating complex interventions.

Methods

The protocol was developed using a systematic literature review, expert consensus, and validation. A 15-member multidisciplinary expert consensus group used the Delphi consensus method to refine the protocol, achieving a consensus threshold of 80%. Validation involved a seven-member expert group evaluating the protocol’s relevance, clarity, comprehensiveness, and appropriateness using a five-point Likert scale. The Content Validity Index (CVI) was calculated.

Results

The NICP-TBI incorporated interventions from clinical practice guidelines and evidence-based protocols. It established clear treatment goals and interventions for each parameter concerned such as airway, ventilation, systemic and cerebral perfusion, intracranial pressure (ICP), sedation, seizure prophylaxis, fluid management, nutrition, infection prophylaxis, temperature management, venous thromboembolism (VTE), blood glucose management, positioning, tapering treatment and family involvement in care. The protocol used a tiered structure adaptable to resource-limited settings. The Delphi process reached a 99.2% final consensus after five rounds, and the CVI was determined to be 1, confirming high validity.

Conclusions

The NICP-TBI provides a structured, evidence-based framework for managing moderate and severe TBI. The protocol’s consensus-driven development, tiered approach in management, and expert validation ensure its applicability across diverse clinical settings, including resource-limited environments. This study highlights the importance of expert consensus and validation in developing effective critical care protocols.

## Introduction

Traumatic brain injury (TBI) is a significant global public health concern associated with high mortality and disability rates, particularly among younger populations [1,2]. The primary injury and secondary brain damage that develops during treatment influence patient recovery. Consequently, developing a well-structured treatment protocol is crucial for improving outcomes [3-5].

Recent advancements in critical care have emphasized maintaining stable cerebral perfusion pressure [6], managing intracranial pressure (ICP) [7], and incorporating multimodal monitoring [8]. However, consistent application of these principles in clinical practice remains challenging, often due to disparities in healthcare resources, technological availability, staffing, and the diversity of TBI cases [9]. Evidence suggests that structured treatment protocols can enhance patient outcomes and reduce mortality rates in TBI management [10-13].

This study aims to develop a neuro-intensive care protocol for TBI (NICP-TBI) to improve patient outcomes. The Medical Research Council (MRC) framework for developing and evaluating complex interventions served as the conceptual basis for this study [14]. This report outlines the development process, including the protocol’s main components, validation, and results.

## Materials and methods

The development and validation of the NICP-TBI followed a structured approach guided by the MRC framework. Key steps included a systematic literature review, adaptation of interventions to local contexts, expert consensus development, and protocol validation. This study was conducted at Tellicherry Cooperative Hospital, Thalassery, Kerala, and Sri Ramachandra Institute of Higher Education and Research, Chennai, Tamil Nadu.

Literature review and evidence synthesis

A systematic literature review was conducted to gather and analyze evidence on neuro-intensive care protocols (NICPs) for TBI management. Searches of PubMed, Cochrane Library, and MEDLINE (Medical Literature Analysis and Retrieval System Online) were performed using keywords such as “traumatic brain injury,” “neuro intensive care,” “TBI management,” “protocols,” and “guidelines.” The review included theoretical models such as neuroprotection, neuroplasticity, the biopsychosocial model, the Monro-Kellie doctrine, and cerebral perfusion [6,8,15-17].

Clinical practice standards in hemodynamic monitoring, respiratory management, sedation, analgesia, temperature control, nutritional support, and seizure prevention were also reviewed [6-8,18-25]. Additionally, key clinical guidelines, including those from the Brain Trauma Foundation (BTF), the American Association of Neurological Surgeons (AANS), the American College of Surgeons, the Neurocritical Care Society, the National Institute for Health and Care Excellence (NICE), and the European Brain Injury Consortium were analyzed [26-29]. Studies testing the effectiveness of these guidelines on functional outcomes after TBI, as well as research on the development and validation of prognostic indicators of TBI, were also reviewed [26-29]. The NICP included a section on family involvement in the care of TBI patients during the neurocritical care stay [30].

Protocol adaptation and local context

In accordance with the MRC framework [14], the researchers adapted interventions to meet local healthcare needs and resource constraints. The goal was to demonstrate that high-quality care and effective patient outcomes are achievable, even in resource-limited settings, by using comparable alternatives to advanced technologies [3-30].

Expert consensus development

A 15-member NICP Expert Consensus Group (NICP-ECG) was established to review and refine each protocol component. The group included neurosurgery, neuro-intensive care, neurology, emergency medicine, surgery, orthopedics, physical medicine, and neuro-nursing specialists, each with over five years of TBI management experience. Using the Delphi consensus method, the researchers achieved agreement on all protocol components, with an 80% threshold for consensus.

Validation and refinement

A seven-member NICP Validation Expert Group (NICP-VEG) was formed, comprising neurosurgeons, neurologists, intensivists, emergency physicians, surgeons, and neuro nurses with over five years of TBI management experience. The validation group assessed the protocol for relevance, clarity, appropriateness, comprehensiveness, and essentiality. Feedback was collected using structured evaluation tools, leading to the refinement and finalization of the validated NICP-TBI. The content validity index was calculated using IBM SPSS Statistics for Windows, Version 23.0 (Released 2015; IBM Corp., Armonk, New York, United States).

## Results

Evidence supporting neuro-intensive care management of TBI

The development of the NICP-TBI drew upon current evidence, including comparative effectiveness studies, neurophysiological theories, practice standards, and guidelines. The Monro-Kellie doctrine and theories of cerebral autoregulation helped in formulating interventions for managing cerebral perfusion pressure and ICP. Additional interventions, such as multimodal monitoring to prevent secondary brain injuries, controlled ventilation, hyperosmolar therapy, and the use of sedatives and analgesics to lower metabolic demands, were based on neuroprotection and neuroplasticity principles, as well as the metabolic needs of the injured brain.

The BTF guidelines served as the foundation for designing protocol components. Advanced monitoring techniques and early surgical interventions were developed with input from the AANS, the Neurocritical Care Society, and the American College of Surgeons Trauma Quality Program. Recommendations from the European Brain Injury Consortium guided neuroimaging criteria. The multidisciplinary framework of NICP-TBI incorporated principles from the NICE guidelines.

Protocol design and modeling

The MRC framework for developing and evaluating complex interventions provided the conceptual structure for designing NICP-TBI. The protocol established clear treatment goals for TBI management. The protocol formulated interventions to manage essential components including airway and ventilation, systemic and cerebral circulation, ICP, CPP, sedation, seizure prevention, infection control, fluid balance, nutrition, and patient positioning.

A tiered approach to treatment was integrated, emphasizing continuous monitoring and methods for tapering off treatments. The protocol identified therapies currently not recommended for TBI and included strategies for involving family members in patient care within the neuro-intensive care unit.

Formation of the NICP-ECG

A multidisciplinary expert group was constituted to guide protocol development. Experts actively involved in TBI care were contacted by phone to explain the study process and invited to participate. Those who consented to join the group received formal invitation letters and agreement forms for documentation.

Development of protocol guidelines

The Delphi method was employed to achieve consensus among the 15-member expert group. The draft NICP-TBI, accompanied by evidence-based notes, levels of recommendations, and references for each intervention, was circulated for feedback. Experts were asked to provide suggestions and modifications within a 15-day review period.

Following each Delphi round, researchers compiled the expert feedback, annotated the suggestions with evidence notes, and prepared those for subsequent rounds of review. Feedback remained anonymous throughout the process. After five Delphi rounds, consensus exceeded the preset threshold of 80%, with a final agreement of 99.2%. Details of consensus percentages for individual components are presented in Table 1.

**Table 1:** The consensus outline of NICP-TBI NICP: neuro-intensive care protocol; TBI: traumatic brain injury

Section	Final Vote (%)
Treatment Goals	100
TBI-Specific Basic Management	98
Tier One	98
Tier Two	99
Tier Three	98
Tapering Therapy	100
Contraindicated Treatments	100
Family Involvement in Care	100

Validation by the NICP-VEG

A seven-member NICP-VEG validated the finalized protocol. This group comprised two neurosurgeons, three critical care specialists, one neurologist, and one neuro nurse, all with extensive experience managing TBI patients.

Validation process

Members of the validation group received formal invitations, participant information sheets, and informed consent forms. Feedback was requested within 15 days using a five-point Likert scale to evaluate the protocol’s relevance, clarity, comprehensiveness, appropriateness, and essentiality. The Content Validity Index (CVI) was calculated and determined to be 1, confirming the high validity of the NICP-TBI.

## Discussion

The development and validation of the NICP-TBI employed a systematic approach to enhance the care of TBI patients. Following the MRC framework for designing and evaluating complex interventions ensured the protocol’s rigor and contextual relevance [14].

While adhering to globally recognized standards, the protocol addressed practical challenges encountered in resource-limited settings. The BTF guidelines provided a scientific basis for selecting interventions, particularly emphasizing a tiered approach to ICP management. Noninvasive measures, such as head elevation and sedation, were recommended before advancing to hyperosmolar therapy and decompressive craniectomy [26].

The AANS guidelines prioritized early detection and timely interventions to prevent further neurological damage. The NICP-TBI incorporated these principles by including routine neuroimaging and structured pathways for intervention within the first 24 hours of admission [27]. Similarly, multimodal monitoring and temperature management strategies from the European Society of Intensive Care Medicine and Neurocritical Care Society guidelines were integrated to prevent cerebral hyperthermia, minimize metabolic demands, and reduce brain tissue hypoxia [29].

Guidance from the NICE shaped the protocol’s approach to early imaging, systemic optimization, and oxygenation management. Both the NICE guidelines and NICP-TBI emphasized maintaining arterial oxygen (PaO2) and carbon dioxide (PaCO2) levels within safe thresholds to mitigate secondary brain injury risks [28]. Additional guidelines, such as Collaborative Head Injury and Guidelines (CHIRAG) [3] and the Tas et al. study [6], highlighted sedation, analgesia, and seizure prophylaxis to reduce metabolic demands and prevent ICP spikes. These interventions were seamlessly integrated into the NICP-TBI.

The tiered structure of the protocol also aligned with Chestnut et al.’s Consensus REVised Imaging and Clinical Examination (CREVICE) Consensus Guidelines, which recommended risk-benefit-based approaches to ICP management [23]. This structure provided flexibility, enabling adaptation to varying resource levels.

The Delphi consensus method was employed during the protocol development phase, involving multidisciplinary experts in neurocritical care. Consensus was achieved on all protocol components after multiple rounds of review and feedback. This iterative, expert-driven process ensured the protocol’s alignment with best practices and practical feasibility.

A structured validation process further strengthened the protocol’s clarity, feasibility, and applicability. Experts systematically reviewed its components using a five-point Likert scale, yielding a high CVI. This validation supports the protocol’s adequacy and potential for implementation in diverse clinical settings.

Strengths and limitations

The MRC framework provided a systematic and evidence-based methodology for protocol development. Engaging a multidisciplinary team with extensive TBI management experience enhanced the protocol’s feasibility and effectiveness. The expert-driven validation process also ensured its relevance and applicability across various healthcare contexts.

However, the study had limitations. The protocol focused solely on intensive care unit management of TBI, excluding initial trauma care at the scene, emergency department management, and rehabilitation. Furthermore, pediatric TBI, a critical population, was not addressed. Future directions include testing the protocol’s impact on functional outcomes in TBI patients and exploring the use of artificial intelligence for protocol implementation.

## Conclusions

This study developed and validated the NICP-TBI, a structured approach for managing moderate and severe TBI. Grounded in recent evidence-based research, neurophysiological theories, and clinical guidelines, the protocol bridges the gap between research and clinical practice. The MRC framework guided its development, while the Delphi method ensured expert consensus and relevance.

Validation through expert feedback confirmed the protocol’s feasibility and adaptability to diverse healthcare settings, including resource-limited environments. By integrating evidence-based interventions, tiered treatment strategies, and assessment criteria, the NICP-TBI offers a robust framework for improving clinical and functional outcomes. This study highlights the critical role of expert consensus and validation in developing effective protocols for critical care.
